# Gum Guillotine Forceps *vs.* Gum Scissors

**Published:** 1884-02

**Authors:** 


### GUM GUILLOTINE FORCEPS *vs.* GUM SCISSORS.

The number of the Independent Practitioner for November last, gave an account and cut of "Abbott's Scissors" for excising the gum over erupting teeth. The number for January contained a letter from Dr. L. D. Shepard, giving a description of Woodhouse's Gum Guillotine Forceps, an instrument of English origin, devised two years or more ago. A letter from Dr. W. H. Eames, of St. Louis, gives an account of an instrument intended for the same purpose, and not unlike the other two in principle. All three of these seem to have been original devices, but the priority of invention certainly seems to be with the St. Louis instrument. This was, Dr. Eames says, invented by Dr. H. S. Chase, eight or nine years ago, and a description and cut of it were published in the Missouri Dental Journal at the time. Dr. Eames has in his possession one of the instruments, and sends us full drawings and description of it.

In the advertisement of Codman & Shurtleff, of Boston, in this number, will be found a cut of an instrument which they manufacture and have on sale. In answer to a letter of inquiry, they state that they make no claim to originality of invention for their Gum Cutter. It was made from the description of an English instrument, which they had never seen. It more nearly resembles that of Dr. Chase, than that of Woodhouse, or Abbott.

The want of such an instrument has long been felt, and it is a little singular that so many different forms should have been devised by men so widely separated. It indicates that the need of it was as universal as would seem to be the supply.

